# PDLIM4/RIL-mediated regulation of Src and malignant properties of breast cancer cells

**DOI:** 10.18632/oncotarget.27410

**Published:** 2020-01-07

**Authors:** Dmitry Sergeevich Kravchenko, Anna Evgenyevna Ivanova, Elizaveta Sergeevna Podshivalova, Stepan Petrovich Chumakov

**Affiliations:** ^1^Shemyakin-Ovchinnikov Institute of Bioorganic Chemistry of the Russian Academy of Sciences, Moscow, Russia

**Keywords:** RIL, PDLIM4, Src, breast cancer, metastases

## Abstract

RIL/PDLIM4 gene was identified as a tumor suppressor, its expression is frequently altered in various types of malignancies. The product of RIL/PDLIM4 gene is an adapter protein involved in the actin cytoskeleton remolding and assembly of stress fibers crucial for cell motility and epithelial-mesenchymal transition. Although the exact mechanism tethering RIL to cancer development remains unknown some pieces of evidence suggest that RIL may act by suppressing activation of the proto-oncogene tyrosine-protein kinase Src. To further explore this issue we tested how different expression levels of RIL affected the activity of Src in breast cancer cell lines. RIL was ectopically overexpressed in the cell cultures with its relatively low endogenous level, or, otherwise, was downregulated by RNA interference. Whereas we observed no correlation between expression levels of RIL and activity of Src we found that in several cell lines elevated levels of RIL were associated with higher cell migratory activity along with the increased incidence of breast xenograft formation and metastasizing. The obtained data suggest that in some breast cancer models RIL may not act as Src kinase inhibitor, but rather play the role of a potential oncogene that promotes cell motility and contributes to cancer cells spreading.

## INTRODUCTION

RIL gene (reversion-induced LIM domain, also known as PDLIM4: PDZ and LIM domain 4) encodes a highly conserved adapter protein, the member of the ALP/Enigma family [1, 2]. The presence of PDZ and LIM domains enables RIL to act as a scaffold, interacting with actin-associated proteins [2, 3], cytoplasmic signaling molecules [4], and membrane receptors [5]. Since its identification as a potential tumor suppressor [1], RIL has been shown to be aberrantly expressed in various types of malignancies [6–10]. In breast cancer the impaired expression of RIL is associated with clinical parameters of tumors, such as tumor size, cell ploidy, differentiation status, and SPF (S-phase fraction) value [9, 11]. It was proposed that RIL may act as a regulator of the proto-oncogene tyrosine-protein kinase Src, which promotes aberrant growth of breast tumors by stimulating survival, angiogenesis, proliferation, and invasion pathways [12–14]. Within the framework of the existing model, RIL preferentially recognizes active Y419-phosphorylated form of Src and links it with PTPL1 (tyrosine-protein phosphatase 1), which in turn dephosphorylates and inactivates Src [12, 15]. This way, induced expression of RIL maintains Src in an inactive form and inhibits cancer progression.

Conversely, a growing body of data suggests that RIL may also act as an oncogene [16–18]. The most aggressive breast cancer cell lines MDA-MB-231, MDA-MB-436, and BT-474 have high levels of RIL expression concomitant with unfavorable prognostic parameters, such as the increased CD74 and low E-cadherin levels [16]. These effects are not explained by the RIL-mediated attenuation of Src activity and call into question whether this model it universally applicable. To resolve this discrepancy, we studied the association between the level of RIL and several characteristics of breast cancer cell lines. Those included the activity of Src, the cell migration rate, the tumorigenicity, and the incidence of breast cancer formation in a mouse orthotopic xenograft model.

## RESULTS

### Levels of RIL protein do not correlate with the activity of Src kinase in a panel of human breast cancer cell lines

**Figure 1:**
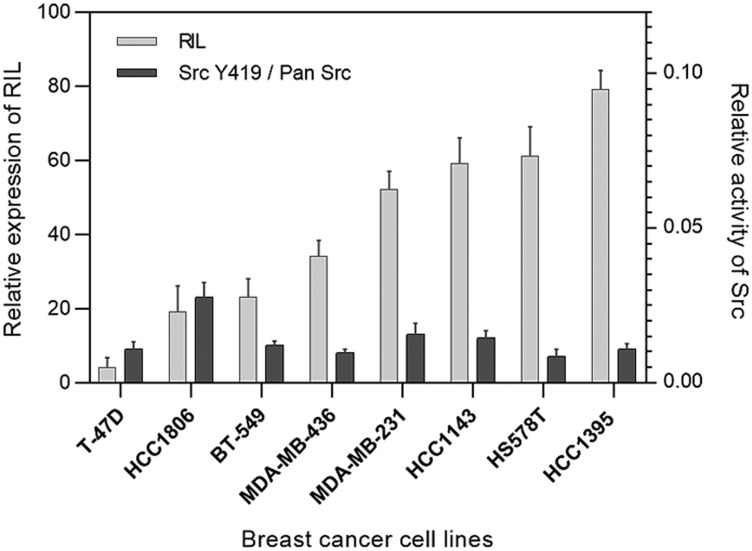
Expression of RIL compared to the activity of Src in breast cancer cell lines; the light bars correspond to the relative expression level of RIL determined by RT-PCR; the dark columns reflect the proportion of active Src (Y419) relative to total Src protein level in the corresponding cells; bars represent standard deviation of the mean.

In order to find a correlation between the level of RIL expression and the activity of Src in the breast cancer model we tested a panel of eight human breast carcinoma cell lines. Expression levels of RIL were measured by qPCR, the proportion of active Src (phosphorylated at Y419) was determined by ELISA (Figure 1).

Quantitative evaluation of the relationship between the two estimated parameters was performed by the Pearson bivariate correlation test. The calculated coefficient stood at -0.172 suggesting no evident correlation between the expression level of RIL and the activity of Src.

The absence of the expected pattern could be explained by the fact that the activity of Src is controlled by numerous tyrosine kinases and phosphatases varying depending on the cell line. The analysis of the panel of different breast cancer cell lines fails to take into account the individual characteristics of each cell culture, which may affect the obtained results.

### Artificial modulation of RIL expression levels in breast cancer cell lines affects the activity of Src kinase

**Figure 2:**
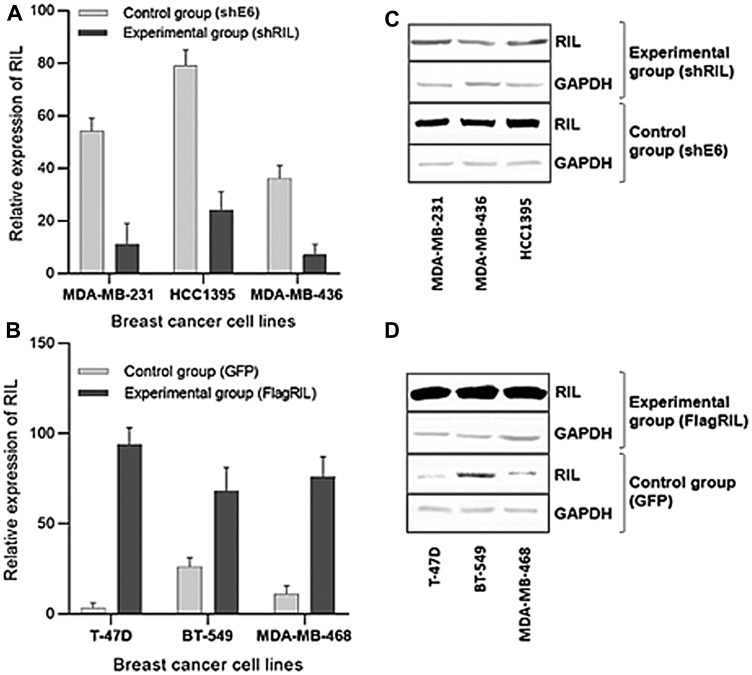
Expression levels of RIL in the established panel of breast cancer cell lines; (**A**, **B**) Real-Time RT-PCR quantitation of RIL suppression (A) and overexpression (B) in the respective cell lines; (**C**, **D**) Western-blot analysis of the suppression (C) or overexpression (D) of RIL, respectively. The bars represent standard deviation of the mean values.

In order to eliminate the impact of cell cultures heterogeneity a panel of breast cancer cell lines with artificially induced and suppressed level of RIL was established. RIL was upregulated in the relatively RIL-deficient cell lines by the introduction of a lentiviral expression construct. To downregulate the expression of RIL in breast carcinoma cell lines with the relatively high levels of endogenous RIL we used RNA interference mediated by the introduction of lentiviral constructs expressing RIL-specific shRNAs (Figure 2). The transgenic cell cultures are listed in Table 1.

**Table 1:** The original and transgenic breast cancer cell lines used throughout the work

The original RIL (+) cell lines, used for suppression of RIL	The obtained transgenic RIL (-) and control cell lines	The original RIL (-) cell lines, used for overexpression of RIL	The obtained transgenic RIL (+) and control cell lines
**HCC1395**	HCC1395 shRIL	**T-47D**	T-47D FlagRIL
	HCC1395 E6		T-47D RFP
**MDA-MB-231**	MDA-MB-231 shRIL	**BT-549**	BT-549 FlagRIL
	MDA-MB-231 E6		BT-549 RFP
**MDA-MB-436**	MDA-MB-436 shRIL	**MDA-MB-468**	MDA-MB-468 FlagRIL
	MDA-MB-436 E6		MDA-MB-468 RFP

**Figure 3:**
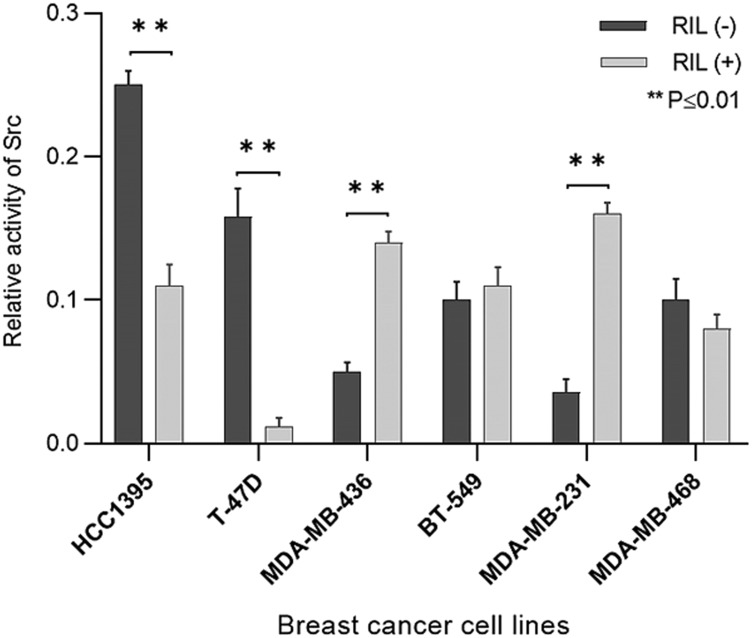
The effect of RIL expression switching on Src activity in breast cancer cell lines; columns reflect the proportion of active Src (Y419-phosphorylated) relative to pan Src; bars represent standard deviation of the mean.

The created panel of transgenic breast cancer cell lines was used to measure the effect of suppression and overexpression of RIL on the activity of Src (Figure 3).

According to the results, the influence of RIL on Src activity varies distinctly between cell lines. While for the T47D and HCC1395 cell cultures the low level of RIL correlates with the considerable inactivation of Src, its activity in BT549 and MDA-MB-468 does not significantly respond to RIL overexpression. Meantime, in MDA-MB-231 and MDA-MB-436 cells the high level of RIL is associated with the increased Src kinase activity, demonstrating the positive correlation between the studied parameters.

### The migratory activity of the RIL (+) and RIL (-) breast cancer cell lines

The observed RIL-dependent activation of proto-oncogene Src may increase the malignant properties of cells, providing evidence for the oncogenic activity of RIL. To test this effect, we compared the phenotypic characteristics of breast cancer cells within the established panel.

**Figure 4:**
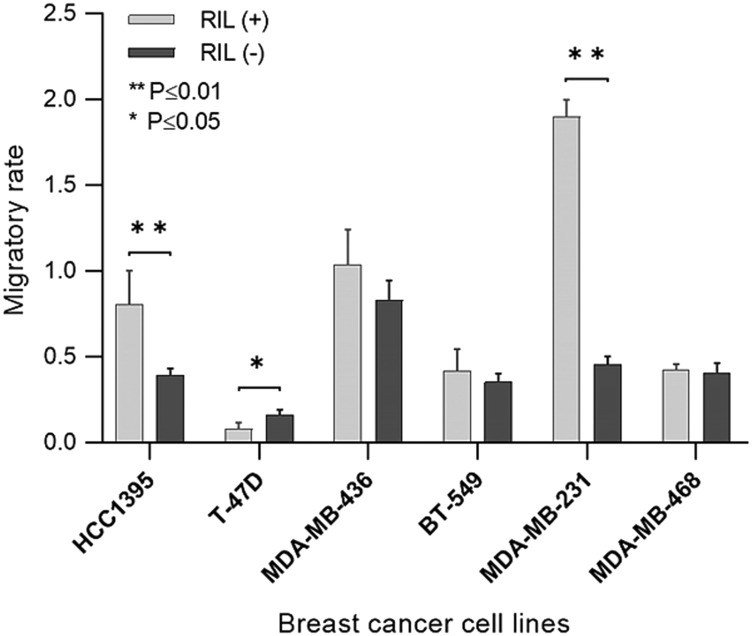
The migratory activity of the transgenic RIL (+) and RIL (-) cells throughout the panel of breast cancer cell lines. The bars represent a standard deviation of the mean.

Migratory activity is considered among critical parameters that determine the aggressiveness of the tumor. The migration level of the RIL (+) and RIL (-) transgenic breast cancer cell lines was evaluated by the Boyden Chamber assay (Figure 4).

According to the data obtained, in two (MDA-MB-231 and HCC1395) of the six cell lines the expression of RIL demonstrates a statistically significant association with the increased migratory activity. The other two cell cultures (MDA-MB-436 and BT-549) show a similar trend. In these cell lines RIL acts as an oncogene stimulating the invasive behavior, which *in vivo* could favor a metastatic spread of cells and a poor outcome of the disease.

### *In vivo* evaluation of oncogenic activity of RIL

Since the oncogenic activity of RIL could be detected *in vitro*, we attempted to confirm the observed effect *in vivo* by evaluating the impact of RIL on tumor formation in the orthotopic xenograft mouse model. The transgenic cells derived from MDA-MB-231 demonstrating the most pronounced positive correlation of RIL expression with the cell migration rate were used within this experiment (Figure 4).

**Figure 5:**
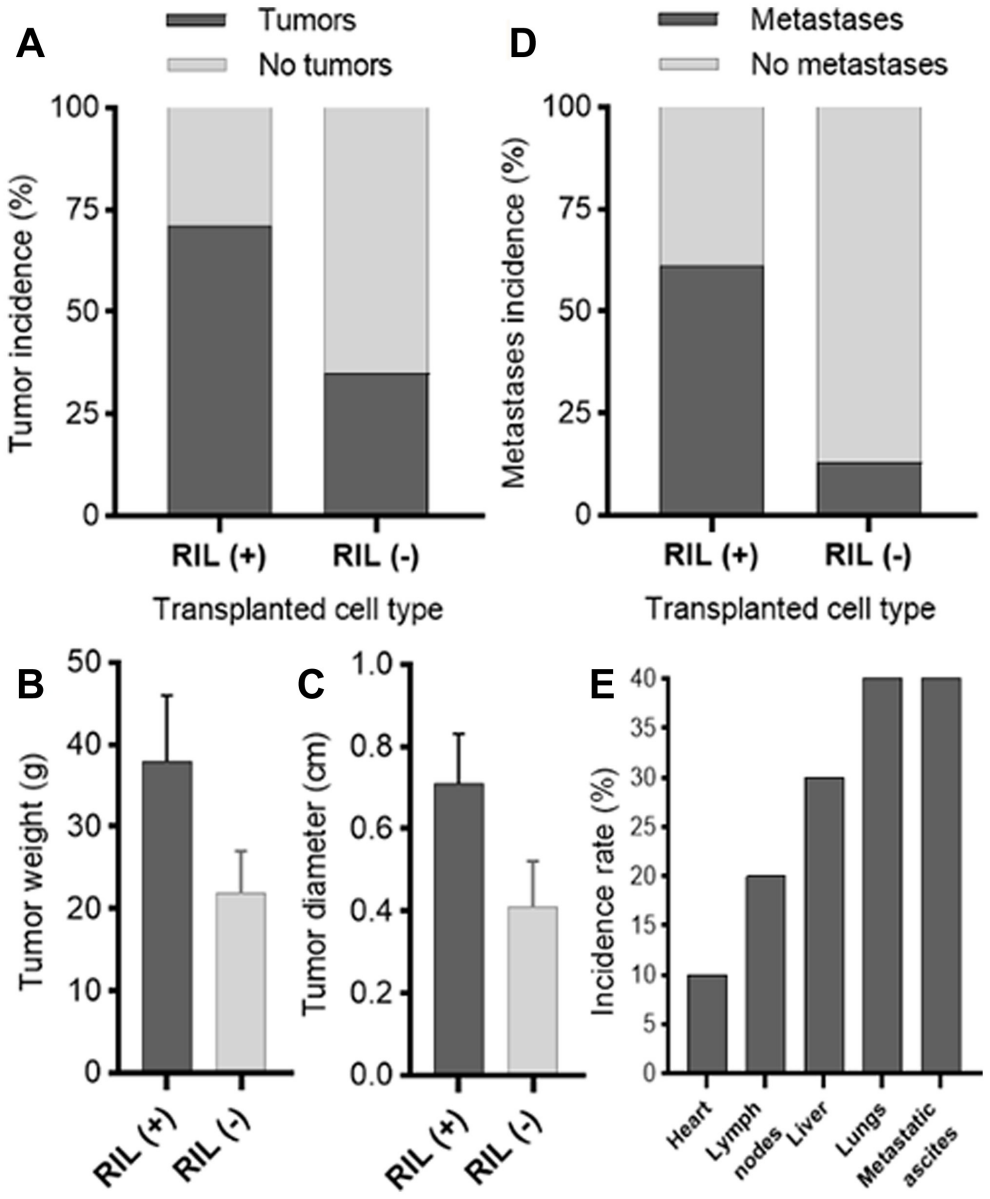
Comparative analysis of the effect of RIL expression on tumor formation (the results for the MDA-MB-231/shRIL and MDA-MB-231/shE6 lines); (**A**) the percentage ratio of the formed tumors in each of the groups; (**B**) average weight of tumors in RIL (+) and RIL (-) groups; (**C**) average diameter of tumors in RIL (+) and RIL (-) groups; (**D**) the rate of detected metastases to the total number of tumors in each group as assessed by RT-PCR; (**E**) the relative incidence of metastases by organ.

The analysis of the xenografts has revealed that RIL-positive cells formed tumors with the approximately one and a half fold larger average diameter and weight than those observed for the RIL (-) group (Figure 5B, 5C). RIL status also affected the tumor formation rate, as RIL (+) tumors occurred more frequently in a statistically significant manner (the value of Fisher’s exact test was 0.0337, *P* < 0.05, Figure 5A). The phi coefficient describing the association between the level of RIL and the tumorigenicity of cells, was 0.346, which, according to the recommendations of Rea and Parker [19], suggests a weakly positive relationship between the studied parameters.

A similar effect was observed for the rate of metastases formation (Figure 5D, 5E). RIL (+) tumors disseminated at more than four times higher rate than RIL (-) tumors. The phi coefficient (ϕ = 0.444) suggests a relatively strong association between the level of RIL and the metastatic spread. These findings are consistent with the *in vitro* results obtained for the correspondent breast cancer cell culture (Figure 4), where the high level of RIL expression was associated with a four-fold increase in the migratory activity.

## DISCUSSION

In the present study, we measured the relationship between expression levels of RIL, phosphorylation status of Src, and phenotypic parameters characterizing malignant phenotype, namely the migration activity of breast cancer cells *in vitro* along with tumorigenicity and metastatic spread in the mouse xenograft model.

As previously it was suggested by Zhang et al. [12] that RIL acts as a tumor suppressor by controlling the dephosphorylation of Src at Y419, we tested this model directly by modulating expression levels of RIL. We found no definite correlation between the endogenous levels of RIL and the activation status of Src in both the case of shRNA-mediated suppression and overexpression of RIL in breast cancer cells. However, in the MDA-MB-231 and MDA-MB-436 cell lines high level of RIL was associated with the increased Src kinase activity and also with the higher cell migratory rate. These findings suggest that in certain cell cultures RIL may contribute to the malignant phenotype acting as a dominant oncogene. In agreement with the suggestion, we found that the downregulation of RIL in MDA-MB-231 cells substantially reduced the tumorigenicity in the mouse xenograft model that affected both tumor incidence and size. There was also a significant reduction in the metastatic spread of tumors to different organs of tumor-bearing mice.

It’s worth noting that the positive correlation between the migratory rate and the expression level of RIL was observed not only for the cultures for which the effect of RIL-dependent Src activation was previously recorded (MDA-MB-231, MDA-MB-436), but also for HCC1395 cell line (Figure 4) which showed an inverse correlation of RIL expression and Src kinase activity (Figure 3). These results suggest that the relationship between RIL and the processes controlling the epithelial-mesenchymal phenotype is not limited to the regulation of Src activity, pointing to the existence of alternative RIL-dependent mechanisms involved in breast cancer formation.

This assumption is consistent with the results of our previous study, which revealed that RIL overexpression correlated with the unfavorable prognostic parameters of tumors, namely an increased CD74 level, reduced expression of E-cadherin, and inactivated intracellular tumor suppressor protein Scribble. Although the RIL-dependent mechanisms of tumor formation remain elusive, RIL may be involved in breast cancer development through the CD74-mediated mechanism typical of malignant cells [16] where increased expression of CD74 could lead to a mislocalization and inactivation of Scribble and loss of E-cadherin.

Along with the previous evidence of RIL being a tumor suppressor, the present results reveal its more ambiguous role in breast cancer development. As a scaffold protein, RIL could participate in a wide range of protein-protein interactions, whereby its contribution to malignant phenotype could be primarily affected by the cellular context and the spectrum of abnormalities accumulated at the preceding steps of carcinogenesis.

Summing up, RIL should not be considered a canonical oncogene or tumor suppressor. The assessment of the role of RIL in cancer development should be carried out taking into consideration the epigenetic features of the tumor or the cell line. Revealing mechanisms by which RIL affects positively or negatively malignant phenotypes of cancer cells could contribute to future personalized approaches to cancer therapy.

## MATERIALS AND METHODS

### Cell culture

Breast cancer cell lines were obtained from the American Type Culture Collection (ATCC, Rockville, MD, USA) and maintained according to the ATCC’s instructions.

### Stable suppression and overexpression of RIL in breast cancer cell lines

The cell lines used for the artificial modulation of RIL expression were selected within a single molecular subtype of breast cancer (triple-negative subtype).

To upregulate RIL the selected cell lines were transduced with the lentivirus expression construct pLM-CMV-FLAG-RIL. The recombinant RIL was fused with the Flag epitope and placed under the control of CMV promoter. As a control, the corresponding cell lines were transduced with a similar lentiviral construct in which RIL was replaced with a Red fluorescent protein (pLM-CMV-RFP).

To downregulate RIL the pLSLPw-shRIL lentiviral construct was used that contained shRNA expression cassette driven by the H1 promoter within the 3′-LTR. As a negative control we used the pLSLPw-shE6 construct that expressed a shRNA specific for the human papillomavirus E6 protein absent in the human genome.

The corresponding genetic constructs were introduced into breast cancer cell lines by lentiviral transduction (Table 1).

### Lentivirus production and transduction of breast cancer cells

To obtain the lentivirus vector particles, HEK293T cells were co-transfected with four plasmids: the lentivirus vector itself, two helper plasmids (pGag1 and pRev2), and pVSV-G. The transfection was performed via the liposome method with the use of LipofectAmine and Plus-reagents (Invitrogen) according to the conditions provided by the manufacturer. The medium containing recombinant virions was collected from the transfected cells and used to infect the target breast cancer cells.

### Western blotting

Proteins from whole-cell lysates were separated by 10% SDS–PAGE and analyzed by Western blotting for the expression of RIL (1:2000 dilution, sc-166582, Santa-Cruz Biotechnology); GAPDH (1:1000 dilution, ab9483, Abcam) was used as a loading control.

### Real-Time PCR

Quantitative analysis of RIL expression was carried out by TaqMan probe-based Real-time PCR; GAPDH was used as the housekeeping gene. Primers and TaqMan probes were designed using Primer-BLAST tool; a comparative Ct method was used to determine the relative expression level of target genes.

### Cell migration assay

Bipartite Transwell chambers with 8-μm diameter pore (Corning Inc. New York, USA) were used to assess cell migration rate in accordance with the Corning Cell Migration Assay. The migratory activity was estimated as the ratio of the number of cells on the lower side of the membrane to the number of non-migrated cells remaining on the upper side of the membrane; the proposed method allowed to avoid the cell count error which occurs when only the migrated cells were taken into account.

### Src kinase activity assay

RayBio^®^ Phospho-SRC (Y419) and Total SRC ELISA kit was used to assess the activity level of Src, according to the manufacturer’s recommendations. Fluorometric analysis of breast cancer cell lysates was performed at 450 nm using Microplate Reader (Triad LT, Dynex). The relative activity of Src was estimated as the ratio of the optical absorption value obtained for the Phospho-SRC (Y419) to the total amount of endogenous Src.

### *In vivo* xenograft studies

SCID Beige mice (CB17. Cg-PrkdcscidLystbg-J/Crl) were purchased from Charles River Laboratory and kept under conditions of the SPF vivarium at the Institute of Bioorganic Chemistry (IBCH RAS); the experiments were performed in accordance to the guidelines recommended by the Bioethics Commission of IBCH RAS.

Eight-week old female mice were injected unilaterally with 1 × 10^6 cells in 50 µL of PBS into the fourth abdominal fat pad by subcutaneous injection at the base of the nipple. Tumor growth was monitored externally using Vernier calipers for up to 30 weeks; the animals were euthanized when the tumors reached 10% of body weight.

### Detection of metastases

Lymph nodes, liver, heart, and lungs of the each euthanized mice were dissected, homogenized under cryogenic conditions, and used for the extraction of total RNA. The presence of the transgenic human cells (corresponding to the secondary sites of tumor development) in the analyzed organs was defined by RT-PCR. Puromycin-resistance gene presented in the transplanted cell lines (MDA-MB-231 shRIL and MDA-MB-231 E6) was chosen as a marker of metastases.

### Statistical analysis

Statistical differences between groups of nominal, independent samples of small size were determined by Fisher’s exact test; the Bivariate (Pearson) correlation test was used as a measure of strength of the association between two continuous independent variables. All data were analyzed using GraphPad Prism, error bars representing standard deviation of the mean. A *p*-value ≤ 0.05 was considered to be statistically significant.

## Abbreviations

ATCC: American Type Culture Collection
ELISA: the enzyme-linked immunosorbent assay
LTR: Long terminal repeats
PBS: Phosphate-buffered saline
PDLIM4: PDZ and LIM domain 4
PTPL1: tyrosine-protein phosphatase 1
qPCR: quantitative polymerase chain reaction
RIL: reversion-induced LIM domain
shRNA: small hairpin RNA
SPF: S-phase fraction
Y: (Tyr) Tyrosine
